# MicroRNA-146a-5p Negatively Regulates Pro-Inflammatory Cytokine Secretion and Cell Activation in Lipopolysaccharide Stimulated Human Hepatic Stellate Cells through Inhibition of Toll-Like Receptor 4 Signaling Pathways

**DOI:** 10.3390/ijms17071076

**Published:** 2016-07-07

**Authors:** Yuhan Chen, Zhaochong Zeng, Xiaoyun Shen, Zhifeng Wu, Yinying Dong, Jason Chia-Hsien Cheng

**Affiliations:** 1 Department of Radiation Oncology, Zhongshan Hospital, Fudan University, Shanghai 200032, China; cspnr1@126.com (Y.C.); shen.xiaoyun@zs-hospital.sh.cn (X.S.); wuzhifeng2@126.com (Z.W.); dongyinyingfd_1@163.com (Y.D.); 2 Division of Radiation Oncology, Departments of Oncology, National Taiwan University Hospital, Taipei 100, Taiwan; jasoncheng@ntu.edu.tw

**Keywords:** hepatic fibrosis, inflammation, toll like receptor 4, microRNA-146a

## Abstract

Lipopolysaccharide (LPS)/toll-like receptor 4 (TLR4) signaling pathway is demonstrated to be involved in the hepatic fibrosis. MicroRNA (miR)-146a-5p is a key regulator of the innate immune response. The functional significance of miR-146a-5p during the LPS/TLR4 mediated hepatic fibrosis process remains unclear. In this study, we found that TLR4 and α-smooth muscle actin (α-SMA) were up-regulated and miR-146a-5p was down-regulated in human hepatic stellate cell (HSC) line LX2 after LPS stimulation. Overexpression of miR-146a-5p inhibited LPS induced pro-inflammatory cytokines secretion through down-regulating the expression levels of TLR-4, IL-1 receptor-associated kinase 1 (IRAK1), TNF receptor associated factor-6 (TRAF6) and phosphorylation of nuclear factor-kappa B (NF-κB). Knockdown of IRAK1 and TRAF6 also suppressed pro-inflammatory cytokine production by inhibiting NF-κB phosphorylation. In addition, miR-146a-5p mimic blocked LPS induced TRAF6 dependent c-Jun N-terminal kinase (JNK) and Smad2 activation as well as α-SMA production. Taken together, these results suggest that miR-146a-5p suppresses pro-inflammatory cytokine secretion and cell activation of HSC through inhibition of TLR4/NF-κB and TLR4/TRAF6/JNK pathway.

## 1. Introduction

Hepatic fibrosis occurs in response to the chronic liver injury and inflammation induced by viral hepatitis, toxins, autoimmune disease, metabolic disorder and ionizing radiation [1,2]. Hepatic stellate cells (HSCs) play an important role in the development, progression and regression of hepatic fibrosis. With the prolonged stimulation of injurious agents or pro-inflammatory cytokines, HSCs are activated and transformed into myofibroblast-like cells that produce excessive extracellular matrix components (ECM), such as α-smooth muscle actin (α-SMA), leading eventually to liver fibrosis [3]. Inhibition of HSC activation may be an efficient way to reverse fibrosis.

Lipopolysaccharide (LPS), a major component of the outer membrane of Gram-negative bacteria, acts as the prototypical endotoxin interacting with the pattern recognition receptors, such as toll-like receptor 4 (TLR4), which promotes initiating innate immune responses [4]. It is well known that by recognizing LPS, TLR4 mediates signal transduction through the MyD88 or Toll/IL-1 receptor domain-containing adaptor inducing IFN-β (TRIF)-dependent pathway and induces secretion of pro-inflammatory cytokines necessary for the development of effective immunity. LPS/TLR4 signaling pathways had been demonstrated to be involved in the pathogenesis of chronic hepatic inflammation, injury and fibrosis [5]. In addition, those patients with chronic hepatitis and cirrhosis are usually accompanied with elevated serum LPS levels [6,7]. These findings indicated that LPS/TLR4 signaling pathways are strongly associated with hepatic fibrosis.

MicroRNA (miRNA) is a small non-coding RNA molecule with the length ranging from 20 to 25 nt. miRNAs induce translation repression or the cleavage of the target mRNAs by binding to complementary sequences in the 3′ untranslated regions (3′ UTRs) of the target mRNAs [8]. Previous researches have revealed that miRNAs regulate several gene expressions influencing many aspects of cellular biology, such as cellular energy metabolism, growth rate, angiogenesis, invasiveness, metastasis and apoptosis [9,10,11]. It was reported that miR-146a-5p participates in the process of hepatic fibrosis through regulating the proliferation and activation of HSCs [12,13]. However, the functional significance of miR-146a-5p during the LPS/TLR4 mediated hepatic fibrosis process remains unclear. In the present study, we found that miR-146a-5p overexpression suppressed LPS induced pro-inflammatory cytokines secretion and HSC activation through inhibition of TLR4 signaling pathway.

## 2. Results

### 2.1. Expression of Toll-Like Receptor 4 (TLR4), α-Smooth Muscle Actin (α-SMA) and miR-146a-5p in LX2 Cells Stimulated with Lipopolysaccharide (LPS)

**Figure 1:**
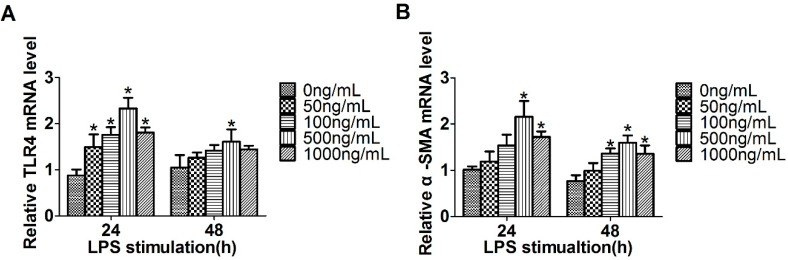

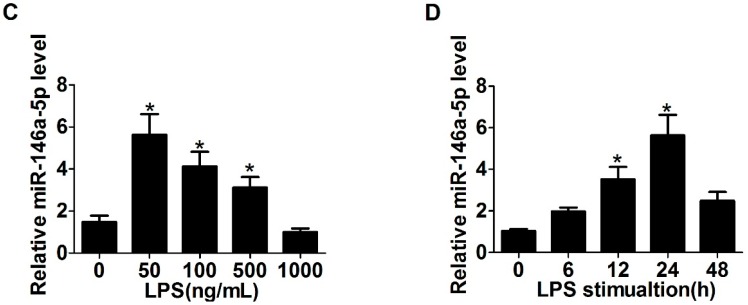
Lipopolysaccharide (LPS) up-regulated toll-like receptor 4 (TLR4) and α-smooth muscle actin (α-SMA) expression and down-regulated miR-146a-5p expression: (**A**,**B**) LX2 cells were treated with different amounts of LPS (0~1000 ng/mL) for 24 or 48 h and then quantitative real-time PCR (qRT-PCR) were used to detect the expression of TLR4 and α-SMA; (**C**) miR-146a-5p expression was examined by qRT-PCR after treatment with different amounts of LPS (0~1000 ng/mL) for 24 h; and (**D**) miR-146a-5p expression was assessed by qRT-PCR after treatment with 50 ng/mL of LPS for the various times (0, 6, 12, 24 and 48 h). * *p* < 0.05 vs. control group.

To investigate whether TLR4 is in response to LPS in LX2 cells, the cells were treated with different concentrations of LPS ranging from 0 to 1000 ng/mL. Compared with the untreated cells, the level of TLR4 increased significantly with 500 ng/mL of LPS stimulation for 24 h (Figure 1A). Meanwhile, the HSC activation marker α-SMA mRNA expression increased in accordance with the TLR4 level in LPS treated LX2 cells (Figure 1B). Our results showed that LPS promotes TLR4 expression and LX2 cell activation and the optimum concentration is 500 ng/mL. We further investigated the involvement of miR-146a-5p in LPS stimulated HSC conversion. LPS treatment resulted in the decreased expression of miR-146a-5p in a dose-dependent manner by 24 h (Figure 1C). In addition, the miR-146a-5p level reached peak in the presence of 50 ng/mL LPS after 24 h stimulation (Figure 1D).

### 2.2. Overexpression of miR-146a-5p Reduced LPS Induced Pro-Inflammatory Cytokines Secretion

**Figure 2:**
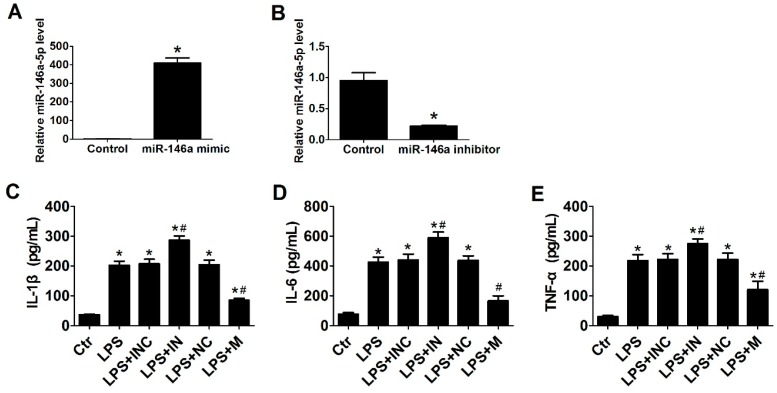
Overexpression of miR-146a-5p reduced the production of interleukin-1 beta (IL-1β), IL-6 and TNF-α in LX2 cells. LX2 cells were transfected with miR-146a-5p mimic or inhibitor for 24 h, followed by incubation with LPS (500 ng/mL) for another 24 h. qRT-PCR showed that the expression of miR-146a-5p in LX-2 cells was significantly elevated by miR-146a-5p mimic (**A**) and decreased by miR-146a-5p inhibitor (**B**). The miR-146a-5p mimic control or inhibitor control served as the corresponding negative control. Overexpression of miR-146a-5p inhibited the production of: (**C**) IL-1β; (**D**) IL-6; and (**E**) TNF-α in LX2 cells after treatment with 500 ng/mL of LPS for 24 h. * *p* < 0.05 vs. blank control group; # *p* < 0.05 vs. the corresponding negative control group. Ctr: blank control; INC: miR-146a-5p inhibitor control; IN: miR-146a-5p inhibitor; NC: miR-146a-5p mimic control; M: miR-146a-5p mimic.

Cells were treated with 500 ng/mL LPS for 24 h in the absence or presence of miR-146a-5p mimic, inhibitor or negative control. Following transfection with miR-146a-5p mimic (Figure 2A) or miR-146a-5p inhibitor (Figure 2B), the expression of miR-146a-5p was significantly up-regulated or down-regulated, respectively. The secretion of interleukin-1 beta (IL-1β), interleukin-6 (IL-6) and tumor necrosis factor-alpha (TNF-α) from LX2 cells were significantly increased after LPS stimulation. After the pretreatment with miR-146a-5p inhibitor in LX2 cells, the production of IL-1β, IL-6, and TNF-α further increased, while overexpression of miR-146a-5p by transfection with miR-146a-5p mimic led to a significant decrease in the production of these cytokines (Figure 2C–E).

### 2.3. miR-146a-5p Inhibited Cytokines Secretion by Down-Regulating the Downstream Genes of TLR4 Signaling Way

**Figure 3:**
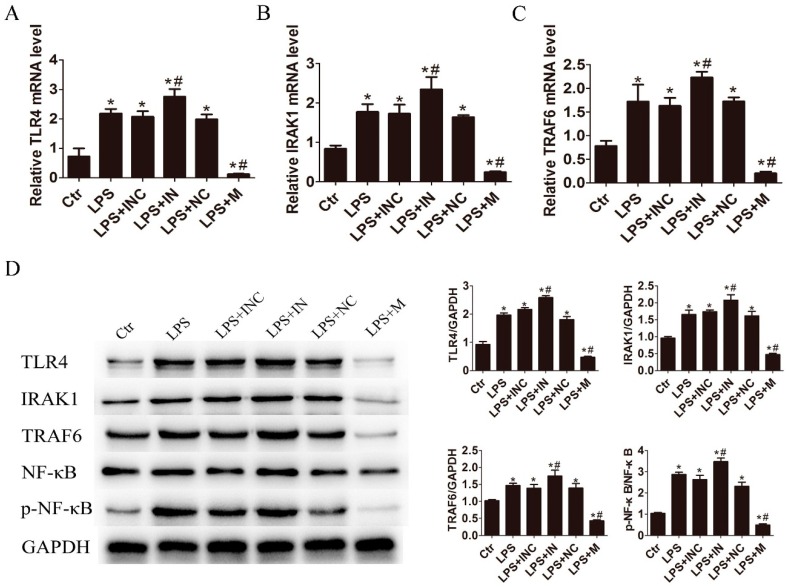
Overexpression of miR-146a-5p inhibited the activation of TLR4/ nuclear factor-kappa B (NF-κB) pathway. After transfection with miR-146a-5p mimic or miR-146a-5p inhibitor for 24 h, cells were exposed to LPS (500 ng/mL) for 24 h. The mRNA expression levels of: (**A**) TLR4; (**B**) IL-1 receptor-associated kinase 1 (IRAK1); and (**C**) TNF receptor associated factor-6 (TRAF6) were reduced by miR-146a-5p mimic and increased by miR-146a-5p inhibitor in LX2 cells; and (**D**) Western blot showed that miR-146a-5p mimic decreased the expression levels of TLR4, IRAK1 and TRAF6 and phosphorylation of NF-κB, which was elevated in miR-146a-5p inhibitor treatment group. * *p* < 0.05 vs. blank control group; # *p* < 0.05 vs. the corresponding negative control group. Ctr: blank control; INC: miR-146a-5p inhibitor control; IN: miR-146a-5p inhibitor; NC: miR-146a-5p mimic control; M: miR-146a-5p mimic.

To investigate the involvement of miR-146a-5p in TLR4 signaling pathway, we analyzed the expression status of TLR4 and its downstream factors associated with pro-inflammatory cytokine production. As compared to the control, the mRNA and protein expression levels of TLR4, IL-1 receptor-associated kinase 1 (IRAK1), TNF receptor associated factor-6 (TRAF6) and phosphorylation of nuclear factor-kappa B (NF-κB) were significantly increased after LPS treatment. Inhibition of miR-146a-5p further enhanced the expression of these key factors, while miR-146a-5p mimic significantly reduced the expression of TLR4, IRAK1 and TRAF6 on both mRNA (Figure 3A–C) and protein levels as well as the phosphorylation of NF-κB (Figure 3D). These data indicate that miR-146a-5p negatively regulates pro-inflammatory cytokine secretion through modulating the TLR4 signaling pathway.

### 2.4. Knockdown of IL-1 Receptor-Associated Kinase 1 (IRAK1) and TNF Receptor Associated Factor-6 (TRAF6) Suppressed LPS Induced Pro-Inflammatory Cytokines Production

**Figure 4:**
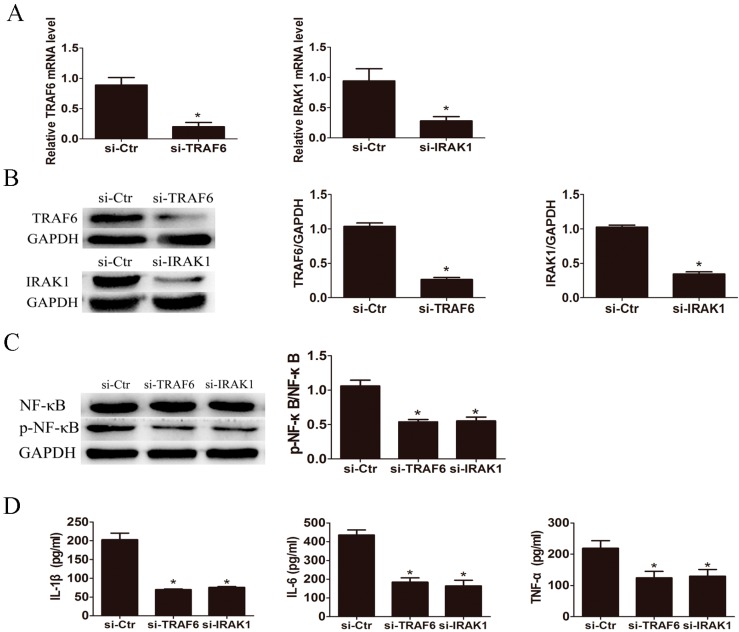
Knockdown of TRAF6 and IRAK1 suppressed the production of IL-1β, IL-6 and TNF-α in LX2 cells. TRAF6 siRNA (si-TRAF6) and IRAK1 siRNA (si-IRAK1) efficiently decreased the mRNA (**A**) and protein expression (**B**) of TRAF6 and IRAK1, respectively. Both TRAF6 and IRAK1 siRNA significantly attenuated the phosphorylation of NF-κB (**C**) and suppressed the production of IL-1β, IL-6 and TNF-α in LX2 cells after treatment with 500 ng/mL of LPS for 24 h (**D**). * *p* < 0.05 vs. control group (si-Ctr).

To verify the influence of TLR4 downstream key factor IRAK1 and TRAF6 on LPS induced pro-inflammatory cytokine production, we knocked down IRAK1 and TRAF6 in LX2 cells by small interfering RNA (siRNA) transfection. The expression levels of IRAK1 and TRAF6 were significantly down-regulated after RNA interference confirmed by qRT-PCR (Figure 4A) and Western blot (Figure 4B). Moreover, respective knockdown of TRAF6 and IRAK1 markedly reduced the phosphorylation of NF-κB induced by LPS in LX2 cells (Figure 4C). Knockdown of TRAF6 and IRAK1 significantly inhibited the LPS-triggered IL-1β, IL-6 and TNF-α production (Figure 4D).

### 2.5. Overexpression of miR-146a-5p Attenuated LPS Induced c-Jun N-Terminal Kinase (JNK) Activation and α-SMA Expression

**Figure 5:**
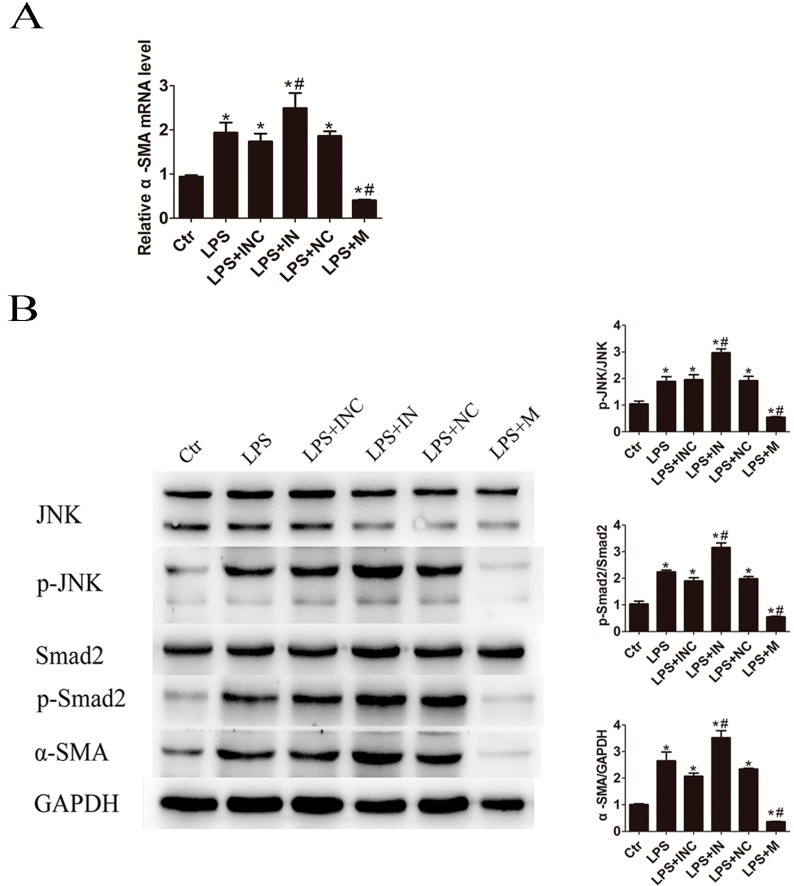
miR-146a-5p mimic attenuated LPS induced JNK activation and α-SMA expression. After pretreatment in the absence or presence of miR-146a-5p for 24 h, LX2 cells were stimulated with 500 ng/mL of LPS for 24 h: (**A**) Overexpression of miR-146a-5p significantly inhibited α-SMA mRNA expression; and (**B**) Western bolt showed that miR-146a-5p mimic significantly decreased LPS induced p-JNK, p-Smad2 and α-SMA expression while miR-146a-5p inhibitor showed the contrary changes. * *p* < 0.05 vs. blank control group; # *p* < 0.05 vs. the corresponding negative control group. Ctr: blank control; INC: miR-146a-5p inhibitor control; IN: miR-146a-5p inhibitor; NC: miR-146a-5p mimic control; M: miR-146a-5p mimic.

Next, the effects of miR-146a-5p on the HSC activation marker α-SMA expression in LPS-treated LX2 cells were studied. The results demonstrated that transfection of miR-146a-5p mimic significantly suppressed α-SMA mRNA and protein expression (Figure 5). Silencing miR-146a-5p by miR-146a-5p inhibitor increased the expression level of α-SMA compared with inhibitor negative control treatment. As demonstrated in Figure 5B, LPS induced α-SMA expression was associated with the activation of c-Jun N-terminal kinase (JNK) and Smad2. Furthermore, we checked if JNK/Smad2 signaling way can be down-regulated by miR-146a-5p. As shown in Figure 5B, pretreatment with miR-146a-5p mimic significantly decreased LPS induced phospho-JNK (p-JNK) and phospho-Smad2 (p-Smad2) expression in LX2 cells compared with the mimic control-transfected cells. Inhibition of miR-146a-5p showed the contrary changes on p-JNK and p-Smad2 expression. 

### 2.6. Knockdown of TRAF6 but Not IRAK1 Inhibited LPS Induced JNK Activation and α-SMA Expression

**Figure 6:**
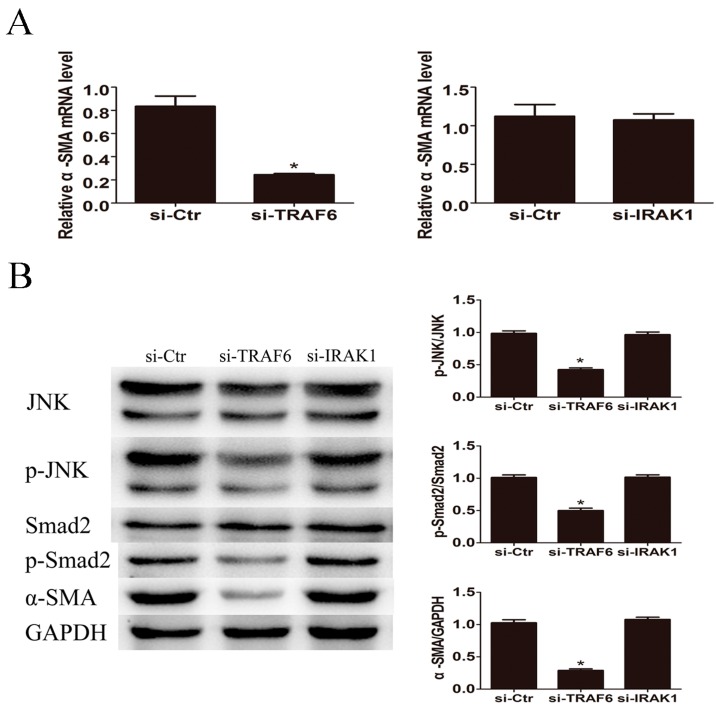
TRAF6 siRNA reduced LPS induced JNK activation and α-SMA expression. After incubation with TRAF6 siRNA or IRAK1 siRNA for 48 h, LX2 cells were treated with 500 ng/mL of LPS for 24 h. (**A**) TRAF6 siRNA but not IRAK1 siRNA decreased the mRNA expression level of α-SMA; (**B**) TRAF6 siRNA but not IRAK1 siRNA attenuated the activation of JNK and Smad2 and decreased the protein expression of α-SMA. * *p* < 0.05 vs. control group.

The mRNA and protein expression levels of α-SMA were significantly reduced by knockdown of TRAF6 but not IRAK1 (Figure 6). Western blot revealed that p-JNK and p-Smad2 expression were down-regulated in response to TRAF6 but not IRAK1 siRNA transfection (Figure 6B). These results indicated that LPS induced HSCs activation through TRAF6 dependent JNK/Smad2 activation.

## 3. Discussion

Hepatic damage induced by viral hepatitis, chronic alcohol consumption, metabolic disorder and ionizing radiation leading to cirrhosis is the result of a complex mechanism. Sustained liver inflammation process mediated by secretion of several cytokines and infiltration of inflammatory cells in the microenvironment of injured liver promotes the progression of liver fibrosis [14]. It is demonstrated that the progression of fibrosis in human hepatic diseases is strongly correlated with the degree of sustained hepatic inflammation [15]. Activation of HSCs, the main fibrogenic cell type in the injured liver, is a key step in liver fibrogenesis. HSCs activation characterized as a transdifferentiation from a quiescent vitamin A-storing cell to a proliferative myofibroblast. The activated HSCs migrate and proliferate at the injured sites, playing a pivotal role in the progressive accumulation of ECM proteins finally leading to the hepatic fibrosis. Emerging evidence has revealed that perpetuating hepatic inflammation contributes to the activation of HSCs [16]. 

TLRs serve as functional link between inflammation and fibrosis in the chronically injured liver. Among the TLR family, TLR4 has been recognized as the most critical toll homolog to activate potent immune responses by LPS stimulation [17]. Accumulating evidence has demonstrated the important role of LPS/TLR4 signal transduction pathway in the activation of HSC and immune cells during the hepatic fibrosis process [18]. In this study, TLR4 was significantly up-regulated after LPS stimulation in LX2 cells. The expression level of α-SMA was elevated in line with the TLR4 status, indicating TLR4 may undergo regulating the activation of LX2. Previous studies showed that miR-146a-5p was down-regulated in the activated HSCs in vitro [12,13]. Similar to their observation, miR-146a-5p expression was down-regulated dose-dependently after stimulation with LPS. Since the HSC proliferation rate may contribute to its activation, we performed CCK8 assay to evaluate the role of miR-146a-5p in regulating LPS stimulated LX2 cells proliferation (Figure S1, Supplementary Materials). The results demonstrated that miR-146a-5p inhibition or overexpression had no effect on LX2 cells proliferation. Therefore, miR-146a-5p inhibition or overexpression was used to investigate the function of miR-146a-5p in LPS stimulated LX2 cells in the following experiments except in the case of cell proliferation.

It is well known that LPS/TLR4 signaling pathway triggers an inflammatory cascade via the recruitment of IRAK1 and TRAF6 to promote NF-κB activation, thus initiating the production of pro-inflammatory cytokines [19]. By means of promoter analysis, miR-146a-5p expression induced by activation of TLR signaling occurs in an NF-κB dependent manner in several kinds of cells [20,21], while elevated expression of miR-146a-5p feedback negatively regulates the pro-inflammatory cytokine secretion and blocks TLR4 signaling pathway by targeting IRAK1 and TRAF6 [22,23]. Moreover, a previous report has shown that TLR4 can be directly down-regulated by miR-146a-5p overexpression since miR-146a-5p enables to bind to the 3′ UTR target sequence of TLR4 determined by a luciferase reporter assay [24]. In the present study, the mRNA and protein levels of TLR4, IRAK1 and TRAF6 in LPS stimulated LX2 cells with miR-146a-5p mimic transfection were significantly decreased, indicating the inactivation of NF-κB signal transduction pathway. The reduced phosphorylation of NF-κB detected by Western blot confirmed this assumption. Our ELISA results showed that increased miR-146a-5p expression significantly attenuated the production of IL-1β, IL-6 and TNF-α in LX2 cells with LPS treatment. The mechanism of anti-inflammatory activity of miR-146a-5p in LPS stimulated LX2 cells is suggested through the inhibition of TLR4/NF-κB mediated pro-inflammatory signaling cascades. TRAF6 and IRAK1 were confirmed to be the unequivocal targets of miR-146a-5p in several studies, thus we further investigated the impact of TRAF6, IRAK1 and their downstream effector NF-κB participating in the pro-inflammatory cytokines secretion. LPS induced NF-κB activation was blocked by preincubation with siRNA specific against TRAF6 and IRAK1, resulting in the reduced secretion of IL-1β, IL-6 and TNF-α. Knockdown of TRAF6 or IRAK1 mimicked the role of miR-146a-5p, further indicating that TRAF6 and IRAK1 are the main functional targets of miR-146a-5p in LPS/TLR4 signaling pathway mediated pro-inflammatory cytokines production.

Several studies have showed that miRNAs are involved in regulating the HSC proliferation, differentiation, and production of ECM. Overexpression of miR-19b inhibits HSC proliferation and suppresses COL1A1 protein levels by targeting growth factor receptor-bound 2 [25]. miR-17-5p promotes HSC activation through inhibiting Wnt inhibitory factor 1 expression and activating Wnt/β-catenin pathway [26]. Transduction of miR-122 in HSCs significantly inhibits the production of mature Col-1 [27]. The effects of miR-146a-5p on liver fibrosis also have been demonstrated in recent investigations. For instance, miR-146a-5p negatively regulates TGF-β1-induced HSC differentiation via decreasing the expression of SMAD4. Another study reported that miR-146a-5p inhibits activation and proliferation of HSCs in the progress of nonalcoholic fibrosing steatohepatitis through suppressing Wnt signaling pathway. In the present study, miR-146a-5p significantly inhibited pro-fibrotic gene α-SMA mRNA expression. A previous study showed that LPS stimulation promotes phosphorylation of JNK and α-SMA expression in HSC-T6 [28]. We also found that LPS promoted the phosphorylation of JNK and Smad2, as well as α-SMA protein production in LX2 cells. miR-146a-5p mimic exerted reduced phosphorylation of JNK and Smad2, together with attenuating protein expression of α-SMA. Additionally, TRAF6 but not IRKA1 siRNA attenuated the JNK, Smad2 activation and α-SMA expression stimulated by LPS, indicating that TRAF6 is critical for LPS induced activation of JNK. Pretreatment with miR-146a-5p mimic or TRAF6 siRNA alleviating LPS induced JNK activation was also reported recently [29]. TRAF6 is an important upstream component of the mitogen-activated protein kinases (MAPKs) pathway and the activation of TRAF6/transforming growth factor-β (TGF-β)-activated kinase-1 signaling complex has been demonstrated to cause the activation of downstream MAPKs [30]. Indeed, transfection with TRAF6 siRNA efficiently inhibited the ERK, p38 and JNK phosphorylation [31,32]. Phosphorylation of the cytoplasmic signaling molecule Smad2 is crucial for canonical TGF-β signaling transduction to regulate fibrosis during liver injury [33]. It is acknowledged that JNK plays a potent role in regulating TGF-β signaling pathway by modulating the phosphorylation of Smad2/3 [34]. Previous research found that JNK in the activated HSCs from CCl4 injured livers directly phosphorylates Smad2/3 and another study also showed that IL-17A enhances the response of HSCs to TGF-β through JNK-mediated Smad2/3 phosphorylation [35,36]. In the present study, miR-146a-5p mediated down-regulation of TRAF6 suppressed the phosphorylation of JNK and Smad2 and attenuated the expression of α-SMA, which might be ascribed to, at least in part, the inactivation of TRAF6/JNK pathway abrogating the induction of pro-fibrotic gene expression. The overall anti-pro-inflammatory and anti-fibrotic effects of miR-146a-5p involved in the LPS/TLR4 mediated hepatic fibrosis need to be further investigated in the animal model in the future study.

## 4. Materials and Methods

### 4.1. Cell Culture

Human hepatic stellate cell line LX2 was obtained from Shanghai Advanced Research Institute, Chinese Academy of Sciences. LX2 cells were cultured in Dulbecco’s Modified Eagle’s Medium (DMEM) (pH 7.4) supplemented with 10 μg/mL streptomycin sulfate, 100 μg/mL penicillin G, and 10% (*v*/*v*) fetal bovine serum (Gibco). The cells were incubated at 37 °C in a 5% CO_2_ humidified atmosphere. For activation of HSCs, LX2 cells were incubated in serum-free media for 48 h before the experiment.

### 4.2. miRNA Inhibitor or Mimic Transfection

The LX2 cells were seeded at a density of 2 × 10^5^/mL in 6-well plate and incubated overnight and then transfected with 50 nM miR-146a-5p mimic or 100 nM miR-146a-5p inhibitor (GenePharma, Shanghai, China) using INTERFERin (Polyplus transfection, Illkirch, France) according to the manufacturer’s instructions. The corresponding negative sequence of mimic or inhibitors (GenePharma) were transfected with the same concentration as controls. For miR-146a-5p mimic, the sequences of oligonucleotides were 5′-UGAGAACUGAAUUCCAUGGGUU-3′ (sense) and 5′-CCCAUGGAAUUCAGUUCUCAUU-3′ (antisense). For miR-146a-5p mimic control, the sequences were 5′-UUCUCCGAACGUGUCACGUTT-3′ (sense) and 5′-ACGUGACACGUUCGGAGAATT-3′ (antisense). The single-stranded RNA sequences were 5′-AACCCAUGGAAUUCAGUUCUCA-3′ for miR-146a-5p inhibitor and 5′-CAGUACUUUUGUGUAGUACAA-3′ for miR-146a-5p inhibitor control. At 24 h after the transfection, cells were harvested or further incubated with LPS (500 ng/mL) for the following experiments.

### 4.3. Quantitative Real-Time PCR

Total RNA, containing miRNA, were isolated from cells according to TRIzol reagent protocol (Invitrogen, Carlsbad, CA, USA). For miRNA analysis, the first-strand cDNA was synthesized using the reverse transcriptase with miRNAs-specific stem-loop primer (RiboBio, Guangzhou, China). Quantitative PCR amplification was conducted in a real-time fluorescent measurement system (ABI7500, Thermo Fisher Scientific, Waltham, MA, USA) using the Bulge-Loop miRNA qRT-PCR Starter Kit (RiboBio) according to the protocol provided. The relative expression level of miRNA was normalized to that of endogenous control U6 by using 2^−∆∆*C*t^ method. For the mRNA of TLR4, IRAK1, TRAF6 or α-SMA analysis, the cDNA was synthesized using the Prime Script RT reagent Kit (Takara Bio, Shiga, Japan) and quantitative RT-PCR (qRT-PCR) analyses were performed by using SYBR^®^ Premix Ex Taq™ (Takara Bio) in the real-time detection system (ABI7500). The sense and antisense primers used included the following sequences: TLR4, 5′-AGTTGATCTACCAAGCCTTGAGT-3′ and 5′-GCTGGTTGTCCCAAAATCACTTT-3′; IRAK1, 5′-AGGTTTCGTCACCCAAACATT-3′ and 5′-CGGGCTGTACCCAGAAGGA-3′; TRAF6, 5′-ATGCGGCCATAGGTTCTGC-3′ and 5′-TCCTCAAGATGTCTCAGTTCCAT-3′; α-SMA, 5′-CTATGAGGGCTATGCCTTGCC-3′ and 5′-GCTCAGCAGTAGTAACGAAGGA-3′; and GAPDH, 5′-CTGGGCTACACTGAGCACC-3′ and 5′-AAGTGGTCGTTGAGGGCAATG-3′. The mRNA level of GAPDH was used as an internal control. 

### 4.4. RNA Interference

LX2 cells were transfected with siRNA against IRAK1 or siRNA against TRAF6, and the negative control siRNA by using the INTERFERin (Polyplus transfection) according to the manufacturer’s instructions. The sequences of IRAK1-specific siRNA were 5′-AGUGGUAGACAUGUAGGAGTT-3′ (sense) and 5’-CUCCUACAUGUCUACCACUTT-3’ (antisense). The sequences of TRAF6-specific siRNA were 5′-GGGUACAAUACGCCUUACATT-3′ (sense) and 5′-UGUAAGGCGUAUUGUACCCTT-3′ (antisense). The negative control sequences for IRAK1 and TRAF6 were 5′-UUCUCCGAACGUGUCACGUTT-3′ (sense) and 5′-ACGUGACACGUUCGGAGAATT-3′ (antisense). The final concentration of siRNA or negative control used for transfection was 50 nM. Knockdown efficiency was evaluated by qRT-PCR and Western blot.

### 4.5. ELISA

LX2 cells were stimulated with LPS (500 ng/mL) for 24 h after transfection with miR-146a-5p inhibitor, mimic, IRAK1 siRNA, TRAF6 siRNA or negative control. The concentrations of IL-1β, IL-6 and TNF-α in culture supernatants were measured with ELISA kits (R & D Systems, Minneapolis, MN, USA) according to the manufacturer’s instruction. Absorbance was recorded at 450 nm using a microplate reader.

### 4.6. Western Blot

LX2 cells were lysed in cell lysis buffer containing PMSF for 30 min at 4 °C. Lysates were collected by centrifugation at 13,000 rpm for 20 min at 4 °C. Proteins from cell lysates were separated on the SDS-PAGE and transferred onto PVDF membrane (Immobion-P Transfer Membrane, Millipore Corp., Billerica, MA, USA). The membrane was blocked with PBST containing 5% non-fat dry milk for 1 h and further incubated overnight at 4 °C with primary antibodies against TLR4, IRAK1, TRAF6, α-SMA (Abcam, Cambridge, MA, USA), JNK, phospho-JNK, NF-κB p65, phospho-NF-κB p65, Smad2, phospho-Smad2 (Ser465/467) (Cell Signaling Technology, Danvers, MA, USA). After that, the membrane was incubated with horseradish peroxidase (HRP)-conjugated secondary antibodies (Santa Cruz Biotechnology, Santa Cruz, CA, USA) for 2 h at room temperature. All protein bands were visualized by ECL method. Anti-GAPDH antibody was used as loading control. Intensity of each protein band was quantified by Quantity One 4.6.2 software (Bio Rad, Hercules, CA, USA).

### 4.7 Statistical Analyses

Data were provided as the mean ± standard deviation from at least three separate experiments. The results were analyzed by Student’s *t*-test, and one-way analysis of variance (ANOVA). Statistical analyses were performed using SPSS 16.0 software (Chicago, IL, USA). *p* < 0.05 was considered statistically significant.

## 5. Conclusions

In conclusion, our study demonstrated that LPS efficiently induces the TLR4 signal transduction cascade to cause activation of NF-κB, phosphorylation of JNK, and promote pro-inflammatory cytokines and α-SMA production in HSC, while these effects could be negatively regulated by miR-146a-5p. These results indicate that miR-146a-5p plays a critical role in hepatic fibrosis, which might aid in the development of novel therapeutic strategies for hepatic fibrosis.

## Supplementary Materials

Supplementary materials can be found at http://www.mdpi.com/1422-0067/17/7/1076/s1.
