# Supplementary material for: MicroRNA-146a-5p Negatively Regulates Pro-Inflammatory Cytokine Secretion and Cell Activation in Lipopolysaccharide Stimulated Human Hepatic Stellate Cells through Inhibition of Toll-Like Receptor 4 Signaling Pathways
Source: Int J Mol Sci. 2016 Jul 7;17(7):1076. doi: 10.3390/ijms17071076 (PMC4964452; doi:10.3390/ijms17071076)
Supplement: Supplementary file 1 [file ijms-17-01076-s001.pdf]

Supplementary Materials: MicroRNA-146a-5p Negatively Regulates Pro-Inflammatory Cytokine Secretion and Cell Activation in Lipopolysaccharide Stimulated Human Hepatic Stellate Cells through Inhibition of Toll-Like Receptor 4 Signaling Pathways

Yuhan Chen, Zhaochong Zeng, Xiaoyun Shen, Zhifeng Wu, Yinying Dong and Jason Chia-Hsien Cheng

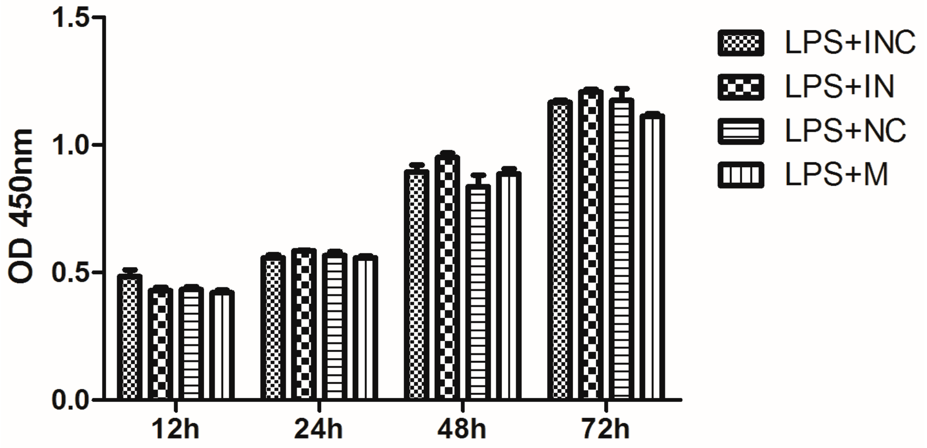

Figure S1. miR-146a-5p inhibition or overexpression had no effect on LPS stimulated LX2 cells proliferation. LX2 cells were transfected with miR-146a-5p inhibitor control (INC), miR-146a-5p inhibitor (IN), miR-146a-5p mimic control (NC) or miR-146a-5p mimic (M) and then stimulated with LPS (500 ng/mL) for various times. The proliferation of LX2 cells at each time point was determined by CCK8 assay.
